# TiO_2_ nanotubes regulate osteo-adipogenic balance through SREBP1 to determine the fate of bone marrow mesenchymal stem cells

**DOI:** 10.1093/rb/rbaf061

**Published:** 2025-06-16

**Authors:** Xiaoxiao Wang, Qiqi Si, Na Yang, Yingying Li, Lingling Tang, Jinsheng Li, Huanghe Zeng, Tingting Li, Song Chen, Tailin Guo

**Affiliations:** School of Life Science and Engineering, Southwest Jiaotong University, Chengdu 610031, China; School of Life Science and Engineering, Southwest Jiaotong University, Chengdu 610031, China; School of Life Science and Engineering, Southwest Jiaotong University, Chengdu 610031, China; School of Life Science and Engineering, Southwest Jiaotong University, Chengdu 610031, China; School of Life Science and Engineering, Southwest Jiaotong University, Chengdu 610031, China; Institute of Biomedical Engineering, College of Medicine, Southwest Jiaotong University, Chengdu 610031, China; School of Life Science and Engineering, Southwest Jiaotong University, Chengdu 610031, China; Department of Health Management Center, Sichuan Clinical Research Center for Cancer, Sichuan Cancer Hospital & Institute, Sichuan Cancer Center, Affiliated Cancer Hospital of University of Electronic Science and Technology of China, Chengdu, 610041, China; Department of Orthopedics of the General Hospital of Western Theater Command, Chengdu, 610083, China; Pancreatic Injury and Repair Key Laboratory of Sichuan Province, The General Hospital of Western Theater Command, Chengdu 610083, China; School of Life Science and Engineering, Southwest Jiaotong University, Chengdu 610031, China

**Keywords:** TiO_2_ nanotubes, SREBP1, fatty acid synthesis, osteo-adipogenic balance, mechanotransduction

## Abstract

Titanium-based materials are commonly utilized in bone tissue repair due to their exceptional physical and chemical properties. Surface modification of titanium dioxide (TiO_2_) nanotubes effectively modulates cellular osteo-adipogenic balance, thereby promoting stem cells osteogenic differentiation. Sterol regulatory element-binding protein 1 (SREBP1), a pivotal transcriptional factor involved in lipid metabolism, plays a significant role in mechanotransduction. Nevertheless, it remains unclear whether SREBP1 also exerts a crucial influence on regulating the differentiation of bone marrow mesenchymal stem cells induced by TiO_2_ nanotubes and its involvement in mechanotransduction during this process. Therefore, this study aimed to investigate the mechanistic role of SREBP1 in cell differentiation induced by TiO_2_ nanotubes. The results demonstrated that TiO_2_ nanotubes exerted regulatory control over SREBP1, enhancing the expression of regulatory factors that induce osteogenic differentiation while suppressing the expression of marker genes associated with adipogenic differentiation. Simultaneously, this regulation inhibited the transcription and translation of pivotal enzymes involved in fatty acid anabolism. Activated by the nanostructure, Lipin1 acted as an upstream target that negatively regulated the expression of SREBP1. The signaling pathway involving Lipin1/SREBP1 was regulated by stress fibers responding to mechanotransduction induced by TiO_2_ nanotubes. Consequently, SREBP1 serves as a critical regulatory factor linking mechanotransduction mediated by TiO_2_ nanotubes and maintaining homeostasis between stem cell osteo-adipogenic differentiation processes. This provides novel insights for designing biomaterials for bone repair.

## Graphical abstract

**Figure:**
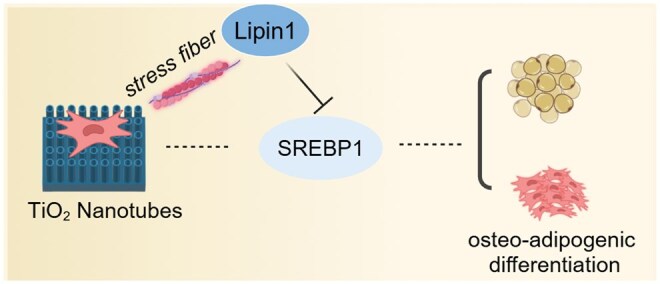

## Introduction

Large segmental bone defects resulting from fractures or bone diseases are challenging to self-heal and require implant-assisted therapies for regeneration. Active bone integration can expedite bone growth within and around the implant [1]. Enhancing the biological integration of the material with the host bone necessitates surface modification of the implant material [2]. Titanium-based bone implants, widely utilized in bone repair due to their excellent corrosion resistance [3], particularly when surface-modified with titanium dioxide (TiO_2_) nanotubes, not only enhance the mechanical properties, biocompatibility and biomimicry of the titanium implants but also feature a porous structure akin to natural bone across various scales, thereby augmenting implant permeability and fostering nutrient exchange [4]. Moreover, TiO_2_ nanotubes play a role in regulating stem cell fate to bolster bone repair. Nanotubes of different sizes enhance calcium deposition in human adipose-derived stem cells, promoting proliferation and osteogenic differentiation [5]. Additionally, they facilitate osteogenesis by mediating crosstalk between macrophages and mesenchymal stem cells (MSCs) under oxidative stress [6]. Titanium implants modified with TiO_2_ nanotubes exhibit elastic deformation under specific mechanical stress, thus, encouraging osteogenic differentiation of bone marrow mesenchymal stem cells (BMSCs) on the nanotube matrix [7]. This nanostructure has the capability to modify the intracellular organization of the actin cytoskeleton, thereby influencing a wide array of cellular dynamics in live cells and impacting stem cell behavior [8]. Nanomorphology also increases the formation of filamentous actin (F-actin) in bone marrow mesenchymal stem cells [9]. F-actin not only acts as a physical structure to support mechanical loading but also participates in other biological behaviors such as signal transduction and gene expression. The reorganization of F-actin and the dynamics of the cytoskeleton play key roles in the cellular response to mechanical signals, which further affects cell morphology, migration and differentiation [10]. Further exploration is necessary to elucidate the specific mechanisms through which TiO_2_ nanotubes regulate stem cell differentiation.

MSCs are multipotent stem cells capable of differentiating into various cell types within diverse microenvironments [11]. However, their directed differentiation into adipocytes and osteoblasts often correlates with abnormal bone remodeling. With aging, the differentiation potential of BMSCs towards osteoblasts declines, while their propensity for adipocyte differentiation increases, contributing to age-related bone loss [12, 13]. Elevated bone marrow adiposity is also evident in osteoporosis patients [14]. Nevertheless, it remains uncertain whether the enhancement of bone repair by TiO_2_ nanotubes correlates with reduced lipogenesis.

Lipid metabolism constitutes a pivotal metabolic pathway in stem cells, playing integral roles in cell membrane architecture, energy metabolism and signal transduction [15, 16]. Fatty acids circulating in the bloodstream serve as signaling molecules, orchestrating stem cell differentiation towards osteoblasts during fracture healing [17]. The Sterol Regulatory Element Binding Protein (SREBP) governs the lipidogenic differentiation of MSCs [18], with its activity diminishing as cellular fatty acid accumulation ensues [19]. SREBP manifests in three isoforms: SREBP-1a, SREBP-1c and SREBP-2. SREBP1 emerges as a pivotal regulatory entity overseeing genes implicated in fatty acid metabolism [20], while also modulating the expression of key lipogenic genes, prominently induced during adipocyte differentiation [21]. Its regulatory mechanism involves the fine regulation of multiple key enzymes and transcription factors, which precisely control the dynamic balance of lipid metabolism through the transcriptional regulatory network [22]. Lipin1 emerges as a recognized regulatory influence on SREBP, operating autonomously of sterol concentrations, thus, revealing an innovative model of steroid-independent SREBP regulation [23]. Lipin1, as a multifunctional regulatory protein, not only possesses phosphatidic acid phosphatase activity, but also acts as a transcriptional coactivator and plays a complex regulatory role in cellular metabolism and differentiation [24]. Nonetheless, the extent to which Lipin1 perceives mechanical cues emanating from the extracellular matrix (ECM), thereby impacting downstream SREBP1 expression and activation, remains elusive, thus, potentially modulating fatty acid metabolism to sway stem cell differentiation.

In this study, the surface of titanium-based materials underwent modification using TiO_2_ nanotubes of varying diameters, with a focus on investigating how these nanotubes regulate the differentiation of BMSCs through SREBP1. Although numerous studies have demonstrated that TiO_2_ nanotubes can significantly promote the osteogenic differentiation of mesenchymal stem cells (MSCs) by modulating cytoskeletal tension and activating classical osteogenic transcription factors such as Runx2, recent research has increasingly recognized the critical role of lipid metabolism in stem cell fate determination. As a central transcriptional regulator of lipid biosynthesis, SREBP1 may play an important role in balancing osteogenic and adipogenic differentiation. Based on this, the present study not only validates the osteoinductive effects of TiO_2_ nanotubes but also further explores whether they regulate BMSC fate through modulation of SREBP1 activity, thereby linking mechanical signals with metabolic regulation. The objective of this research is to provide valuable insights into the equilibrium of BMSCs differentiation within microstructured/nanostructured environments, offer fresh evidence regarding the interaction between mechanical signals from the ECM and cell differentiation, and propose innovative pathways for the advancement of implantable devices featuring novel nanostructures.

## Materials and methods

### TiO_2_ nanotubes

Titanium flakes (3.5 cm diameter, purity > 99%, Haiyuan Research Metals, China) were cleaned with RO water. They were then immersed in an acid wash solution containing 4 wt% HF and 40 wt% HNO_3_, and ultrasonicated for 5 min. Subsequently, they were placed in an anodic oxidation buffer consisting of 0.4 wt% HF and 12.8 wt% H_3_PO_4_ for anodic oxidation (The above chemical reagents are all from Kelong Chemical, China). The anode was made of titanium sheet and the cathode was made of graphite, both parallel to each other. The oxidation process was carried out using a high-voltage programmable DC power supply (BD60120-12, Bona Electric, China) at 5 V (NS5) and 20 V (NS20) for 1.5 hr. After the process, the buffer on the titanium wafers was rinsed off with RO water and the wafers were left to dry naturally. The titanium wafers were then sintered by heating them up to 450°C at a rate of 5°C/min, holding them for 3 hr and allowing them to cool naturally to room temperature. The titanium wafers underwent surface characterisation using scanning electron microscopy (SU5000, Hitachi, Japan) and a contact angle test.

### BMSCs isolation and purification culture

BMSCs were extracted from 1- to 2-week-old male Sprague Dawley rats (Chengdu Dashuo Laboratory Animal Co., Ltd, China) and cultured in low-glycemic medium (90% DEME low-glycemic medium[Gibco, USA] + 10% FBS[BI, Israel] + 1% antibiotics[Sigma, USA]) for 1 day. The medium was then discarded, and the cells were washed with PBS before being added back into the low-glycemic medium for further culture. The culture medium was changed every two days until the cell fusion rate reached 80% under the microscope. Then, the cells were passaged and subsequently cultured in αMEM medium (90% αMEM medium[Gibco, USA] + 10% FBS + 1% antibiotics). The culture medium was changed every 3 days, and BMSCs from the third to sixth generations were collected for subsequent experiments.

### Alizarin red staining

Titanium sheets (Flat, NS 5, NS 20) were placed in 6-well platesand inoculated with BMSCs (5 × 10^4^ cells/well). The osteogenic induction medium (50 μM ascorbic acid, 100 nM dexamethasone, 10 mM sodium β-glycerophosphate; The above reagents are from Alfa, USA) was replaced when the cell fusion rate reached 70–80%. After 21 days of culture, the cells were fixed with 4% paraformaldehyde (Kelong Chemical, China) and stained with 1% alizarin red staining (ARS) solution (Solarbio, China). Observations were made using a stereomicroscope (SG-700, SAGA).

### Alkaline phosphatase staining

Titanium sheets (Flat, NS 5, NS 20) were placed in 6-well plates and inoculated with BMSCs (5 × 10^4^ cells/well). The osteogenic induction medium was replaced after the cell fusion rate reached 80%. After 14 days of culture, the cells were fixed in 4% paraformaldehyde for 30 min. Alkaline phosphatase staining was carried out using the BCIP/NBT Alkaline Phosphatase Chromogenic Kit (Beyotime, China), and the results were observed with a stereomicroscope.

### ORO staining

Titanium sheets (Flat, NS 5, NS 20) were placed in a 6-well plates and inoculated with BMSCs (1 × 10^5^ cells/well). After the cell fusion rate reached 100%, they were cultured for 2 days. The lipid-forming induction solution I (DMEM low-glycemic medium, 10% fetal bovine serum, 1 μM dexamethasone, 0.5 mM IBMX [Solarbio, China], 10 μg/ml insulin[Solarbio, China]) was then added and cultured for another 2 days. Subsequently, the lipid-forming induction solution Ⅱ (DMEM low-glycemic medium, 10% foetal bovine serum, 10 μg/ml insulin) was replaced and cultured for 2 days. Afterward, the cells were incubated in a 6-well plate for one day and then cultured for one week in the same plate with 10% fetal bovine serum and 10 μg/ml insulin. After 14 days, lipogenesis was observed using a stereomicroscope following staining with the Oil Red O Staining Kit (Biosharp, China) as per the instructions.

### Cytoskeleton staining

Titanium sheets (Flat, NS 5, NS 20) were placed in 6-well plates after inoculation with BMSCs (2 × 10^4^ cells/well). After 7 days of incubation, fix with 4% paraformaldehyde. Add 1 ml of PBS configured with 0.1% Triton X-100 (BioFroxx, Germany) and incubate for 10 min at room temperature. Add 200 μl of Phallotoxins (Bioscience, China) and stain for 30 min at room temperature away from light. Add 2 μg/ml of DAPI (Solarbio, China), and stain for 5 min at room temperature away from light. Observed with a fluorescence microscope (BX53FL, Olympus, Japan) and photographed.

### Real-time quantitative polymerase chain reaction

Titanium sheets (Flat, NS5, NS20) were placed in 6-well plates after inoculation with BMSCs (3 × 10^5^ cells/well) for 7 days. Cellular RNA was extracted using Trizol. cDNA was reverse-transcribed according to the instructions of the Reverse Transcription Kit (ThermoFisher, USA). cDNA was used as a template for a three-step reaction using SYBR (Biosharp, China). The transcript levels of the target genes were obtained by Ct(2-ΔΔCT) calculation method using Gapdh as an internal reference. The primer sequences are shown in Table 1.

**Table 1.** Primer sequences

Gene	Forward primer （5′-3′）	Reverse primer （5′-3′）
*Gapdh*	AGACAGCCGCATCTTCTTGT	ATCCGTTCACACCGACCTTC
*Opn*	AGCAATGAGGTTCGTGTCTCT	TATGCCGCTATGTGCCATCT
*Runx2*	CAGCAGCTTTGCAGGAAGTG	CCCTCCCTAGTGAGAGCCTT
*Ocn*	CCAGATGAGGAACGGTTGGG	GGGGCTGTCTGGGATTGAAC
*Alp*	GGGCCTGCTCTGTTTCTTCA	CTGAGATTCGTCCCTCGCTG
*Pparg*	GAGATCCTCCTGTTGACCCAG	TCCATCACAGAGAGGTCCACA
*Adipoq*	TGTATGGGGAAGGGGACAAC	CGCCTGTTCTTTGATTCTCGG
*Fabp4*	AGAAGTGGGAGTTGGCTTCG	ACTCTCTGACCGGATGACGA
*Cebpa*	AAGTGTCCCCACCCCTAGTT	CCCTTCTCCACGAACTCACC
*Lipin1*	AAAAAGTTCACAAGCAGGGACC	CTCCTTCACCGTCACAAACAC
*Srebp1*	GAGGTGTGCGAAATGGACG	CCTGTGTCTCCTGTCTCACC
*Acc1*	CCCCACAGCCCATTACACAT	TCACTGACACGAGTGAAGGC
*Scd1*	CAAGCCTACCTACTCACTGCC	GTCACAAATAACCGCCCCACA
*Fasn*	GCTTGGTGAACTGTCTCCGA	GTGAGATGTGCTGCTGAGGT
*Ldlr*	CATTTTCAGTGCCAACCGCC	TGCCTCACACCAGTTTACCC

### Transfection of small interfering RNA to reduce gene expression

Find the transcript ID of the target gene via NCBI (https://www.ncbi.nlm.nih.gov/). design the sequence using the small interfering RNA (siRNA) design webpage (Invitrogen Block-iT RNAi Designer [thermofisher.com]) and perform Blastn validation to prevent there being off-target. Gene-specific siRNAs were synthesised by DynaTech Biotechnology (Chengdu, China). The siRNAs were transfected into cells using Lipofectamine RNAiMAX (ThermoFisher, USA) according to the instructions. Two days after transfection, samples were collected. During subsequent ARS, Alkaline phosphatase staining (ALP) and Oil Red O (ORO) staining, BMSCs inoculated on titanium sheets with different micro-nano morphologies were 1 × 10^4^ cells/well, 2 × 10^4^ cells/well and 1 × 10^5^ cells/well, respectively. siRNA sequences are shown in Table 2.

**Table 2.** Names and sequences of siRNA

Gene	Target sequence （5′-3′）
si*Lipin1*	AGGACATGTTTCCCATAGAGATG
si*Srebp1*	TCCTCTATCAATGACAAGATTGT

### Western blotting

BMSCs (3 × 10^5^ cells/well) were inoculated on titanium slices of different morphology by group and protein was lysed after 7 days of incubation. One-hundred twenty microlitre of RIPA lysate, 1% broad-spectrum protease inhibitor and 1% PMSF were added to each well. Protein concentration was determined after protein extraction according to the BCA kit instructions. Add 5× loading buffer (all the above reagents are Boster, USA) 95°C water bath for 10 min. Total protein extract (30 μg) was separated by 10% (w/v) SDS-polyacrylamide gel electrophoresis and transferred to a PVDF membrane. The membrane was closed with 5% skimmed milk for 2 hr. The membrane was incubated with primary antibody dilution overnight at 4°C, washed five times with TBST for 5 min each time and the secondary antibody was incubated at room temperature for 1 hr. After the TBST wash, the membrane was exposed in the darkroom using X-Ray film. Put the membrane into the cassette and suck off the excess TBST, add the prepared ECL luminescent solution, turn off the red light and turn on the red light when there are faint fluorescent bands discernible by the naked eye. Absorbent paper to remove excess luminescent liquid, plus the appropriate size of the film, the pressure box exposure for about 30S. The film was taken out and put into the developing solution for 1–3 min, and after the appearance of bands, it was put into the fixing solution for 2 min. After the exposure of the target protein, remove the PVDF membrane, remove the primary and secondary antibodies according to the instructions of Stripping buffer (Beyotime, Shanghai, China), and repeat the steps of closure as described above. Antibody incubation ratios: SREBP1 (1:5000), FASN (1:5000), GAPDH (1:10 000), β-actin (1:5000), the above antibodies were obtained from Abcam, China; Goat Anti-Rabbit IgG (1:10 000), Goat Anti-Rabbit IgG (1:10 000), the above antibodies were obtained from Beyotime, China (10 000), the above antibodies were from Bioss, China. Antibody incubation ratios: SREBP1 (1:5000), FASN (1:5000), GAPDH (1:10 000), β-actin (1:5000), the above antibodies were obtained from Abcam, China; Goat Anti-Rabbit IgG (1:10 000), Goat Anti-Rabbit IgG (1:10 000), the above antibodies were from Bioss, China.

### Statistical analysis

Data were analysed using GraphPad Prism 8.0. *t*-test was used for comparison between two groups, and Tukey's multiple comparison test and ANOVA were used for data comparison between multiple groups. *P* < 0.05 was taken as statistically significant difference between data.

## Results

### Surface characterisation of TiO_2_ nanotubes

**Figure 1.**
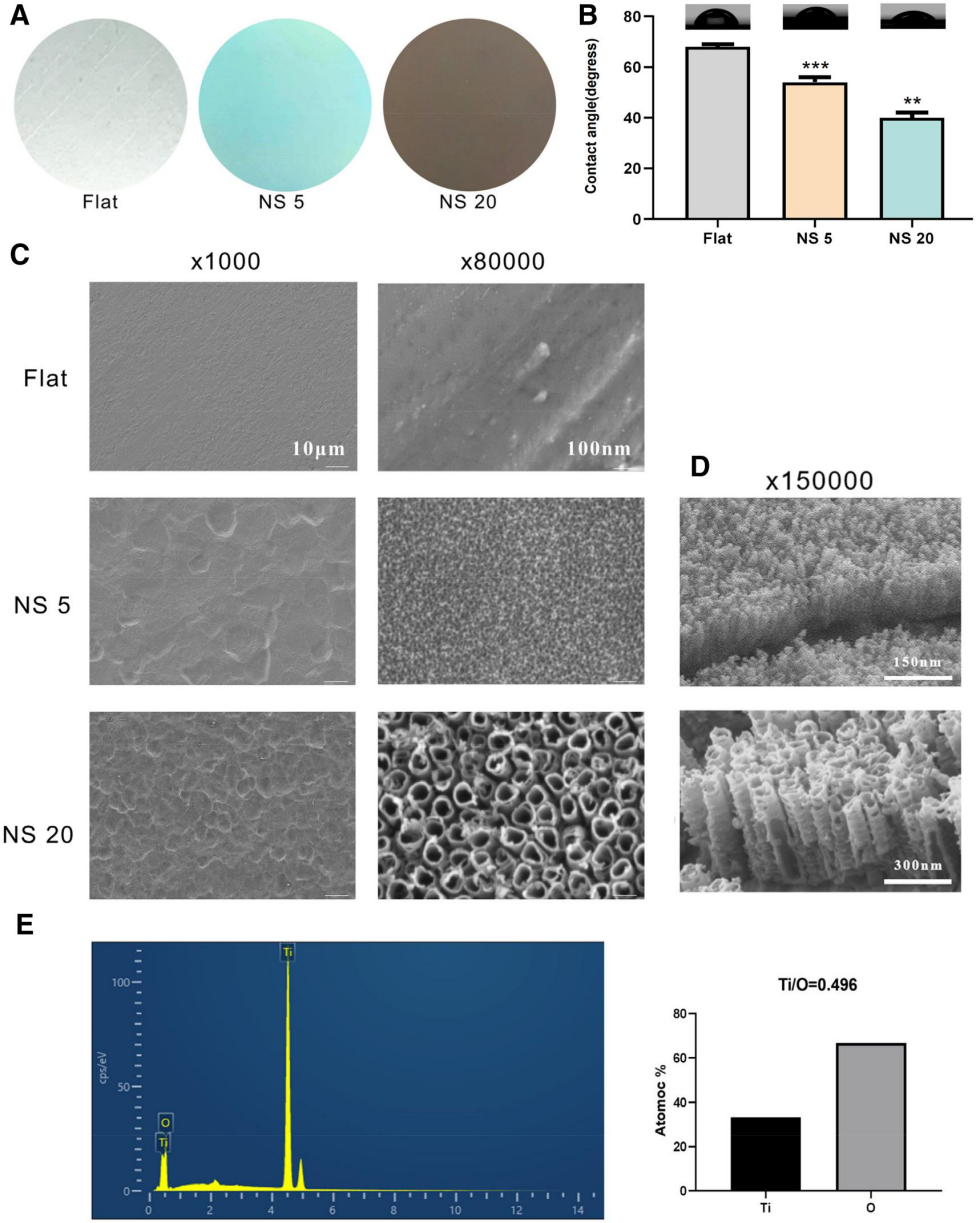
Surface characterisation of titanium flakes after anodic oxidation at different voltages. (**A**) Samples of 3.5 cm diameter titanium flakes after anodic oxidation at different voltages (flat: smooth titanium flakes; NS5: TiO_2_ nanotube arrays prepared at 5 V; NS20: TiO_2_ nanotube arrays prepared at 20 V); (**B**) contact angle test; (**C**) surface morphology of titanium samples observed by scanning electron microscope (SEM), left side magnified 1000× with scale bar of 10 μm, right side magnified 80 000× with scale bar of 100 nm; (**D**) cross-section morphology of titanium samples observed by scanning electron microscope (SEM); (**E**) energy spectroscopic chemical elemental composition analysis.**P < 0.01. ***P < 0.001*, n* = 3.

In this study, the surface of smooth titanium sheets was directly modified through anodic oxidation to enhance their bone integration performance [25, 26]. The surface morphology of the materials was observed using a scanning electron microscope, which showed that the nanotubes of NS 5 had a diameter of about 30 nm and those of NS 20 had a diameter of about 100 nm, and the unanodised Flat did not possess nanotubes as a control. At 1000× magnification, the surface of Flat exhibits smooth characteristics with almost no obvious structural features. In contrast, the anodized NS 5 and NS 20 display tiny particles and uneven structures, with the surface structure of NS20 being more complex. At 80 000× magnification, the smooth titanium sheet maintains its smooth surface characteristics, while the NS5 and NS20 samples reveal a more uniform microstructure, with the shapes and arrangements of the pores becoming more distinct (Figure 1C). The cross-sectional depths of the nanotubes were approximately 130  and 480 nm, respectively (Figure 1D). Therefore, the refracted light wavelengths were different, resulting in a silvery metallic sheen for Flat, a blue colour for NS 5 and a golden colour for NS 20 (Figure 1A). And after contact angle detection analysis, it was found that NS 20 had a smaller water contact angle compared to NS 5, and both were significantly lower than the Flat group (Figure 1B). This indicates that the nanotube materials with different tube diameters are more hydrophilic. The chemical elemental analysis of the energy spectrum showed that the ratio of titanium element to oxygen element on the surface of the material after anodic oxidation was 1:2, which confirmed that they were TiO_2_ nanotubes (Figure 1E). In conclusion, this study proved that the material with TiO_2_ nanotubes was successfully prepared and the morphology is conducive to increasing the contact area with the surrounding tissues and improving the biocompatibility, which can be used as a substrate material for the subsequent experiments. In our previous research, Live/Dead cell staining and Cell Counting Kit-8 (CCK-8) assays demonstrated the excellent biocompatibility of titanium sheets with varying micro-nano surface architectures [27].

### TiO_2_ nanotubes regulate differentiation of BMSCs

**Figure 2.**
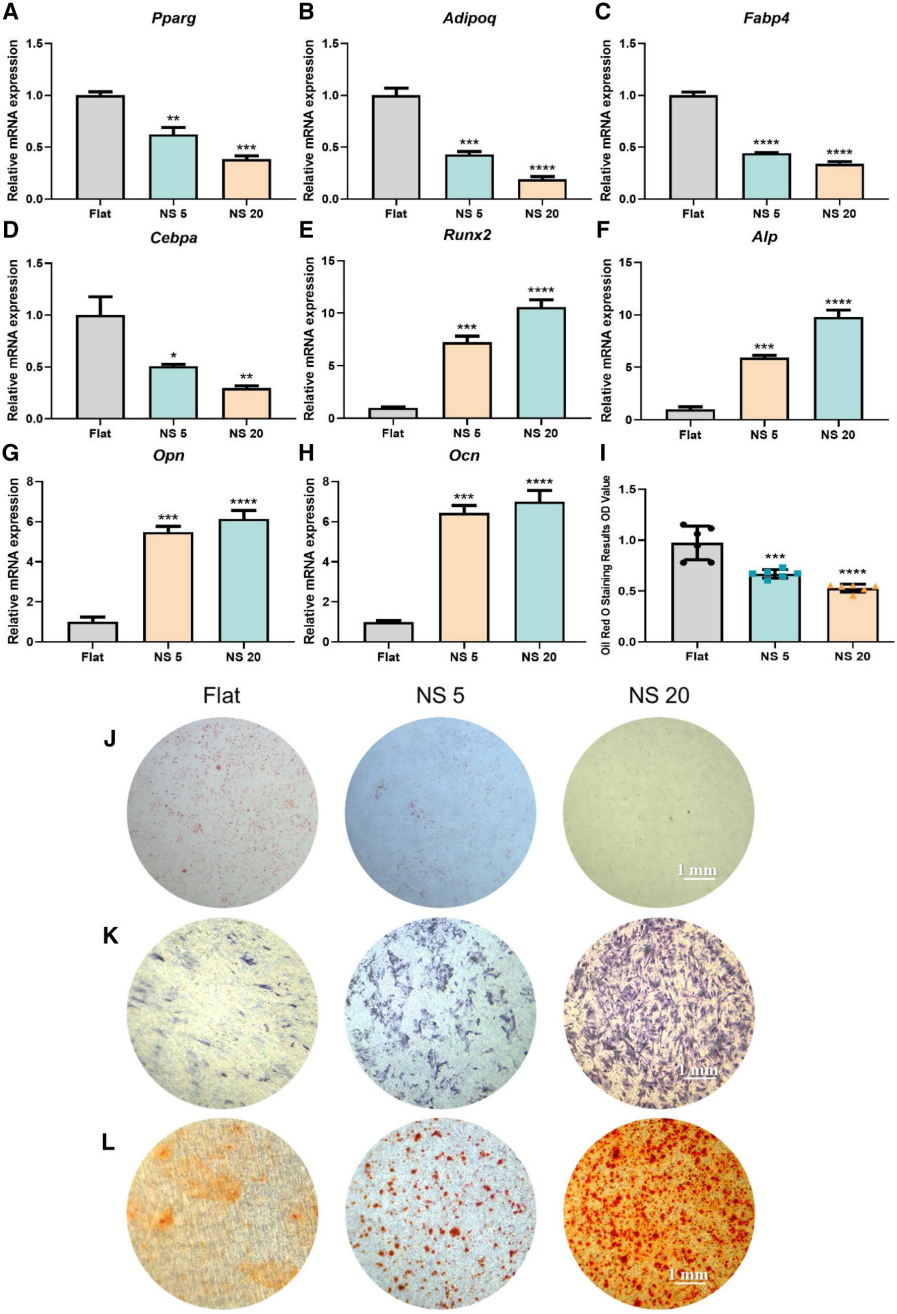
TiO_2_ nanotubes regulate BMSCs differentiation. The expression levels of genes related to the regulation of adipogenic differentiation were detected by RT-qPCR: (**A**) *Pparg*, (**B**) *Adipoq*, (**C**) *Fabp4* and (**D**) *Cebpa*; the expression levels of genes related to the regulation of osteogenic differentiation were detected by RT-qPCR: (**E**) *Runx2*, (**F**) *Alp*, (**G**) *Opn* and (**H**) *Ocn*; (**J**) oil red O staining (**I**) detection of adipogenesis and quantitative analysis, *n* = 6; (**K**) ALP staining to observe ALP enzyme activity; (**L**) ARS staining to observe extracellular calcium nodule generation. Scale bar, 1 mm. *P < 0.05, **P < 0.01, * **P < 0.001, * ***P < 0.0001, *n* = 3. Abbreviations: *Pparg*, peroxisome proliferator-activated receptor gamma; *Adipoq,* adiponectin; *Fabp4*, fatty acid binding protein 4; *Cebpa*, CCAAT/enhancer binding protein α; *Runx2*, Runt-related transcription factor 2; *Alp*, alkaline posphatase; *Opn*, osteopontin; *Ocn*, osteocalcin.

BMSCs are multipotent stem cells that form a regulatory network through key signalling pathways and transcription factors when interacting with various factors in a specific microenvironment. This results in a dynamic equilibrium between lipogenic and osteogenic differentiation in the organism [28]. BMSCs were cultured on titanium sheets with TiO_2_ nanotubes anodized at different voltages to investigate the effect of micro-nano morphology on the lipogenic and osteogenic differentiation of BMSCs. The levels of expression of the lipogenic differentiation marker genes Pparg, Adipoq, Fabp4, and Cebpa were significantly lower in cells inoculated on NS 5 and NS 20 than in cells on Flat (Figures 2A-D). Consistently, the ORO staining results showed that adipogenesis of BMSCs was significantly reduced on the nanotubes compared to Flat (Figures 2I-J). These results suggest that the morphology of TiO2 nanotubes inhibits BMSCs lipogenic differentiation. The expression levels of the osteogenic differentiation regulators Runx2, Ocn, Alp and Opn were induced in cells inoculated on NS 5 and NS 20 at significantly higher levels than those on Flat (Figure 2E–H). The results of ALP staining and ARS staining showed that nanotube morphology increased alkaline phosphatase activity (Figure 2K) and extracellular calcium nodule deposition (Figure 2L) in BMSCs compared with Flat. These results suggest that TiO_2_ nanotube morphology promotes BMSCs osteogenic differentiation.

### Regulation of SREBP1 activation and lipid synthesis metabolism by TiO_2_ nanotubes

**Figure 3.**
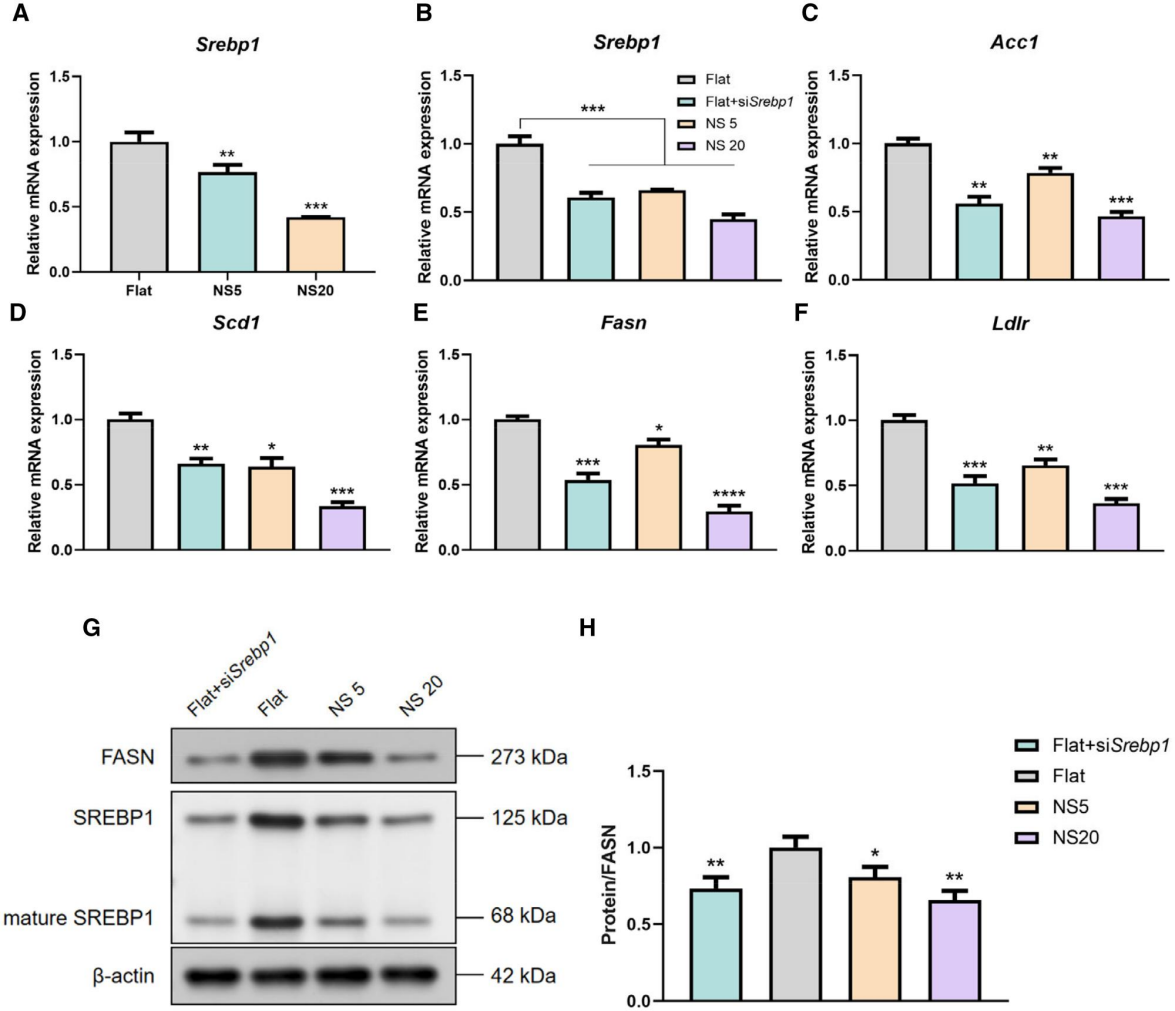
TiO_2_ Nanotubes suppress SREBP1 expression and activation, regulating BMSC lipid synthesis and uptake. (**A**) Detection of Srebp1 expression by RT-qPCR; (**B**) Srebp1 mRNA expression levels in Flat, Flat+siSrebp1, NS5 and NS20 groups; expression levels of key enzymes in lipid metabolism, (**C**) Acc1, (**D**) Scd1, (**E**) Fasn and (**F**) Ldlr, after transfection with siRNA targeting Srebp1, as assessed by RT-qPCR; (**G**) Western blot analysis of FASN protein levels and (**H**) gray value analysis following inhibition of Srebp1 expression. *P < 0.05, **P < 0.01, * **P < 0.001, * ***P < 0.0001*, n = 3, ##P < *0.01, *# ##P < *0.001, *n* = 3. Abbreviations: Acc1, Acetyl-CoA carboxylase 1; Scd1, stearoyl-CoA desaturase 1; Fasn, fatty acid synthase; Ldlr, low density lipoprotein receptor.

Lipids and their derivatives serve as a crucial energy source, and disruption of lipid metabolism can impair osteoblast function, leading to skeletal defects and altering systemic lipid homeostasis. SREBP1, a key regulatory factor in fatty acid *de novo* synthesis, was found to be significantly suppressed in BMSCs by the nanotube morphology compared to the Flat group, as revealed by real-time quantitative polymerase chain reaction (RT-qPCR) analysis (Figure 3A). This indicates the involvement of TiO_2_ nanotubes in regulating SREBP1 expression, potentially impacting lipid metabolism. To investigate the influence of TiO_2_ nanotubes on BMSC lipid metabolism, siRNA was added to the Flat group to interfere with *Srebp1* expression. The results showed that silencing Srebp1 suppressed both its transcription and activation. Downregulation of SREBP1 was observed in the Flat+*siSrebp1* group (Figure 3B). These results indicate successful transfection. Furthermore, to explore the impact of mechanical signals generated by micro-nano morphology on lipid metabolism, gene expression was assessed through RT-qPCR after silencing *Srebp1*. The results indicated that micro-nano morphology inhibits *Srebp1*, thereby suppressing the expression of key enzymes involved in *de novo* fatty acid synthesis, including Acc1, Fasn and Scd1 (Figure 3C–E), and a significant reduction in FASN protein levels (Figure 3G and H). Furthermore, the expression of the key gene Ldlr involved in controlling lipid uptake also decreased (Figure 3F). These findings suggest that TiO_2_ nanotubes can modulate fatty acid metabolism by inhibiting SREBP1 expression.

### TiO_2_ nanotubes regulate BMSCs differentiation by modulating lipid metabolism through SREBP1

**Figure 4.**
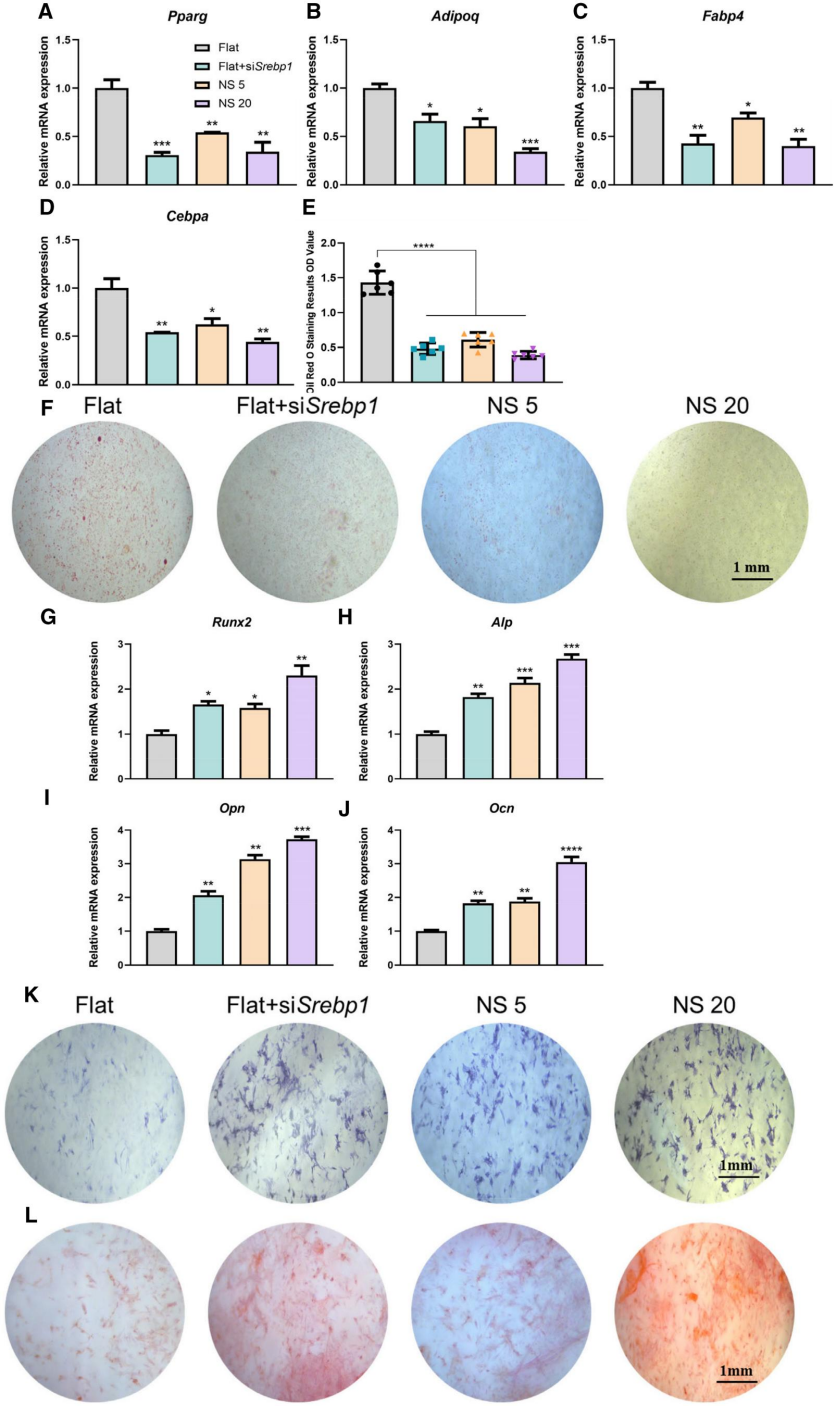
TiO_2_ nanotubes regulate BMSCs differentiation through *Srebp1*. After transfection of siRNA interfering with the expression of *Srebp1*, the expression levels of genes related to the regulation of adipogenic differentiation were detected by RT-qPCR: (**A**) *Pparg*, (**B**) *Adipoq*, (**C**) *Fabp4* and (**D**) *Cebpa*; (**F**) lipogenesis was detected by oil-red O-staining and **(E**) quantitatively analysed, *n* = 6; and the expression levels of genes related to the regulation of adipogenic differentiation were detected by RT-qPCR by interference with expression levels of genes related to the regulation of osteogenic differentiation after *Srebp1* expression: (**G**) *Runx2*, (**H**) *Alp*, (**I**) *Opn*, (**J**) *Ocn*; (**K**) ALP enzymatic activity was observed by ALP staining; (**L**) extracellular calcium nodule generation was observed by ARS staining. Scale bar, 1 mm. *P < 0.05, **P < 0.01, * **P < 0.001, * ***P < 0.0001, *n* = 3.

Lipids can serve as signaling molecules that impact the signal transduction processes of stem cells, thus, influencing their differentiation [29]. Therefore, in the Flat group, SREBP1 expression was downregulated to inhibit fatty acid synthesis, aiming to investigate the relationship between TiO_2_ nanotube-mediated lipid metabolism and BMSCs differentiation. RT-qPCR results revealed that, compared to the Flat group, the downregulation of SREBP1 expression resulted in a consistent decrease in the expression of adipogenic differentiation marker genes, which was similar to the trend observed in the nanotube group (Figure 4A–D). The ORO staining results showed that lipid production also significantly decreased (Figure 4E and F). Conversely, the expression trend of osteogenic differentiation marker genes significantly increased, similar to the nanotube group (Figure 4G–J). The results of ALP staining and ARS staining showed that Alkaline phosphatase activity (Figure 4K) and calcium nodule deposition were also increased (Figure 4L). These findings indicate that TiO_2_ nanotubes inhibit adipogenic differentiation of BMSCs and promote osteogenic differentiation by reducing fatty acid synthesis and uptake through the downregulation of SREBP1 expression.

### Lipin1 responds to the mechanical signals generated by TiO_2_ nanotubes through actin polymerization contraction and inhibits the expression and activation of downstream SREBP1

**Figure 5.**
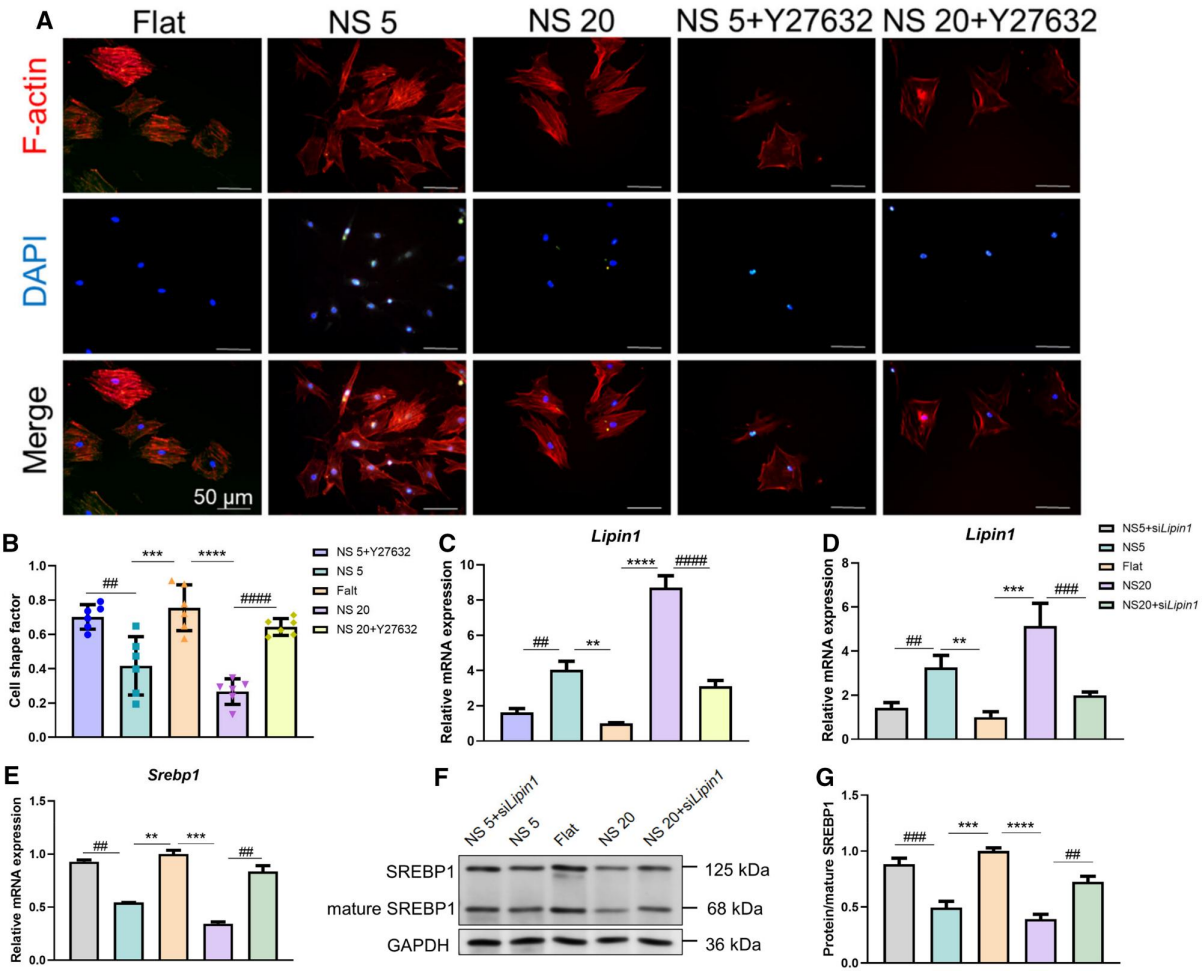
Lipin1 Responds to the mechanical signals generated by TiO_2_ nanotubes and inhibits the expression and activation of downstream SREBP1. (**A**) Cells were labeled with YF dye-conjugated phalloidin and treated with the actin polymerization inhibitor Y27632. Scale bar, 50 μm. (**B**) Cell cytoskeletal development was examined using fluorescence microscopy, and quantitative analysis of cell shape indices was performed, *n* = 6. (**C**) Lipin1 expression after treatment with the Y27632 inhibitor was assessed by RT-qPCR. (**D**) The expression of Srebp1 after inhibiting Lipin1 expression (**E**) was evaluated by RT-qPCR and (**F**) protein levels were analyzed (**G**) along with grayscale values, *n* = 3. **P < 0.01, ***P < 0.001, ****P < 0.0001*, ##P* < 0.01, *###P < *0.001, *####P < *0.0001. Note: Y27632 is a ROCK inhibitor that effectively inhibits actin polymerization; the shape index = cell width/cell length, where a shape index approaching 1 indicates a spherical cell, and a shape index approaching 0 indicates an elongated cell.

The morphological features of microscale and nanoscale materials interact with the ECM components. This interaction may potentially modulate the clustering of integrins and the development of focal adhesions, while also influencing the cellular cytoskeletal structure [30, 31]. Therefore, BMSCs were seeded on titanium substrates with different morphologies, and cell cytoskeleton staining revealed that cells on TiO_2_ nanotubes were more elongated compared to the Flat group (Figure 5A), with cell shape indices mostly below 0.5 and approaching 0 (Figure 5B). This indicates that the morphology of TiO_2_ nanotubes can alter cell morphology, potentially impacting cytoskeletal development and thereby regulating various cellular signaling pathways. Consequently, when the actin polymerization inhibitor Y27632 was added to the TiO_2_ nanotube group, BMSC morphology became more rounded (Figure 5A), and the cell shape index approached 1 (Figure 5B), indicating that BMSCs sense the mechanical signals generated by TiO_2_ nanotubes through actin polymerization contraction. Lipin1 controls lipid synthesis and participates in gene expression pathways related to energy metabolism [32]. Studies have shown that extracellular physical signals inhibit Lipin1 by reducing actin contractility [33]. Therefore, the expression of Lipin1 was assessed by RT-qPCR in BMSCs seeded on titanium substrates with different morphologies. Results showed a significant upregulation of Lipin1 expression in the TiO_2_ nanotube morphology group compared to the Flat group (Figure 5C). This promoting effect was inhibited upon actin polymerization inhibition in the nanotube group (Figure 5C), indicating that the morphology of TiO_2_ nanotubes can enhance Lipin1 expression by promoting actin polymerization. In the known SREBP regulatory context, Lipin1 acts independently of sterol levels [23]. When Lipin1 expression was reduced in the nanotube group (Figure 5D), the activation level and expression of SREBP1 significantly increased (Figure 5E–G), suggesting that Lipin1 senses the mechanical signals generated by TiO_2_ nanotubes to inhibit the expression activation of downstream SREBP1.

## Discussion

The aim of this study was to explore the impact of extracellular mechanical stimuli on the homeostasis of BMSCs differentiation. Cellular behavior is intricately regulated not only by mechanical cues within the microenvironment but also by various biochemical factors that coordinate differentiation processes. Factors such as blood flow, muscle contraction and tissue rigidity can give rise to macroscopic mechanical stimuli, thereby modulating cellular responses at the molecular level. The morphology of cells on flat Ti and layered substrates with micro/nano topography is quite different [34]. Moreover, another investigation demonstrated varying elongation rates of cells on nanotubes depending on tube diameter, with larger diameters correlating to higher elongation rates [35]. Building upon prior research that a too small a diameter leads to insufficient cell adhesion, while too large a diameter reduces the attachment stability of BMSCs, thus, our study fabricated TiO_2_ nanotubes with pore sizes of approximately 30  and 100 nm as substrate materials [36]. The findings indicated that cells cultured on nanotubes exhibited elongated morphologies and denser actin filament networks, particularly pronounced with larger pore sizes. This phenomenon may be attributed to the reduced number of adhesion sites offered by the nanotube pore structure compared to the smooth titanium surface. Consequently, for cells to adhere firmly to the nanotube surface, cell surface adhesion receptors prompt rearrangement and polymerization of the actin cytoskeleton, thereby preserving biomechanical equilibrium within the cell.

MSCs constitute a heterogeneous population of non-hematopoietic stem cells that were initially identified in bone marrow, possessing the capacity to differentiate into mature cells across various mesenchymal tissues, including adipose and bone. The morphological characteristics of MSCs undergo alterations upon interaction with nanotubes [37], thereby enabling their differentiation along distinct pathways [38]. Moreover, when cultured on substrates of varying stiffness, MSCs undergo spontaneous differentiation into adipocytes or osteoblasts, depending on the substrate's rigidity [39, 40]. Notably, investigations have revealed that adipogenic differentiation is suppressed while osteogenic differentiation is significantly enhanced in BMSCs cultured on titanium sheets featuring a pore size of 100 nm, corroborating prior findings [7, 41]. The orchestrated lineage commitment of MSCs plays a pivotal role in preserving the homeostasis of the bone's internal milieu. Dysregulation in MSC differentiation has been implicated in numerous bone disorders [42–45]. Thus, titanium nanotubes emerge as key regulators of differentiation processes, offering potential avenues for ameliorating and rectifying abnormalities associated with bone diseases through reconstruction and repair mechanisms.

Extracellular physical signals impact the mechanical properties of the Golgi apparatus, leading to the inhibition of Lipin1 through a reduction in actomyosin contractility. Phosphorylated adenosine 5′-monophosphate-activated protein kinase (AMPK) can inhibit the mechanistic target of rapamycin complex 1 (mTORC1) signaling pathway by activating tuberous sclerosis complex 2 (TSC2) [46, 47], which in turn regulates Lipin1 transcription [48]. The Ras homologous gene family member A (RhoA) is a small guanosine triphosphatase (GTPase) that governs cytoskeletal dynamics and participates in signal transduction, thereby controlling various cellular functions such as cell survival, migration, adhesion and proliferation [49]. Our study revealed that inhibiting actin polymerization mitigated the upregulation of Lipin1 induced by TiO_2_ nanotube morphology, consistent with prior findings.

**Figure 6.**
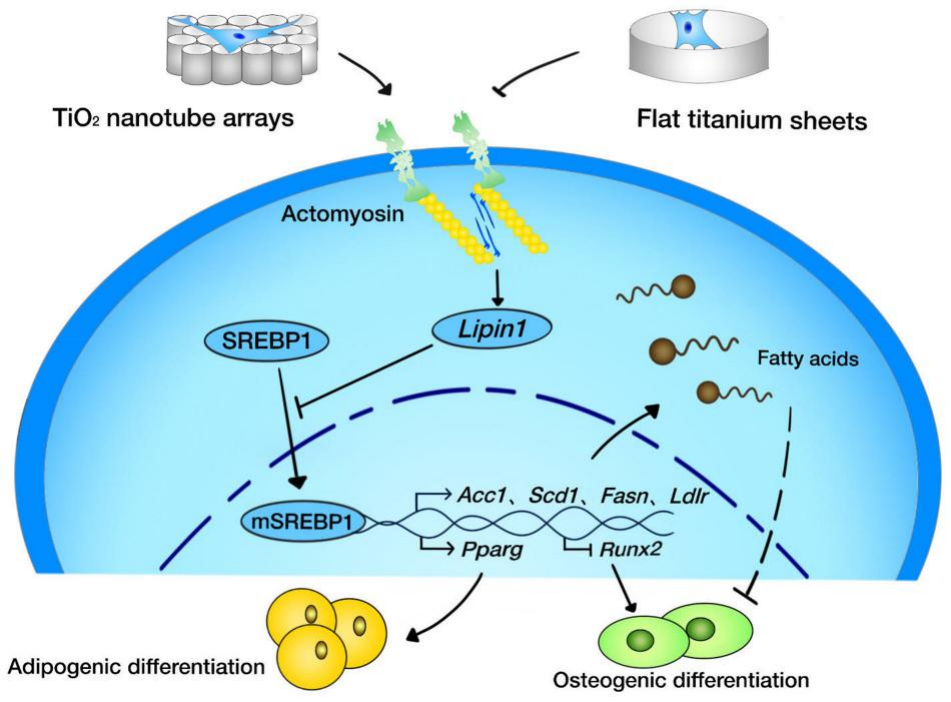
Schematic diagram illustrating the regulation of BMSCs differentiation by TiO_2_ nanotubes via SREBP1.

Additionally, Lipin1 serves as an inhibitor of SREBP1 [50] and can mediate its modulation through mechanical stimulation [33]. Our research revealed that the inhibition of SREBP1 by micro- and nano-morphology was mitigated when Lipin1 expression was diminished in the nanotube group. This phenomenon occurred irrespective of sterol levels, unveiling a novel model of steroid-independent regulation of SREBP. To further illustrate this regulatory mechanism, a schematic diagram is provided that summarizes how TiO_2_ nanotubes influence BMSC differentiation via SREBP1 (Figure 6). BMSCs adhered to TiO_2_ nanotubes respond to mechanical signals, which are transmitted to the cells via actin polymerization and contraction. The transcription of *Lipin1* increases in response to these mechanical signals, leading to inhibition of downstream SREBP1 expression and activation. Activated SREBP1, in turn, stimulates the transcription and translation of key enzymes involved in fatty acid metabolism, thereby facilitating *de novo* synthesis of fatty acids. Furthermore, activated SREBP1 promotes the transcription of adipogenic differentiation marker genes such as *Pparg*, thereby enhancing adipogenic differentiation of BMSCs. Conversely, activated SREBP1 suppresses osteogenic differentiation of BMSCs by inhibiting transcription of osteogenic differentiation marker genes such as *Runx2*.

Lipid metabolism is a fundamental pathway for cellular energy storage and production, playing a crucial role in maintaining metabolic and functional homeostasis in stem cells. Simultaneously, metabolic remodeling guides cellular differentiation. Sterol regulatory element-binding proteins (SREBPs), a family of membrane-bound transcription factors, exert significant influence on cellular metabolism by orchestrating the synthesis of fatty acids, triglycerides and cholesterol [51–53]. It governs the expression of enzymes associated with fatty acid anabolism, thereby impacting the differentiation trajectory of stem cells and modulating the *de novo* synthesis of fatty acids [54, 55]. In the Flat group, reduced Srebp1 expression led to alterations in Acc1, an enzyme that regulates the rate-limiting step in *de novo* fatty acid synthesis [56], as well as FASN, which catalyzes the terminal step of this process [57]. The expression pattern mirrored that of the nanotube group, indicating a downward trend. This underscores the potential of extracellular mechanical cues to influence fatty acid anabolism in BMSCs, thereby linking cellular mechanics to metabolism.

The regulation of SREBP1 by mechanical signaling involves multiple interrelated pathways. For instance, geranylgeranyl pyrophosphate (GGPP), a crucial intermediate in the mevalonate pathway, modulates SREBP1 activity through RhoA isoprenylation and downstream actomyosin contraction. This contractility has been shown to repress the transcription of key lipogenic enzymes, thereby affecting stem cell fate decisions in murine models [58]. Typically, lipid anabolism regulated by SREBP1 is promoted under cytoskeletal tension, whereas energy stress via AMPK activation can inhibit this process to preserve metabolic homeostasis [59, 60]. However, recent findings suggest that the relationship between cytoskeletal tension and SREBP1 activity is context-dependent. Specifically, Romani et al. demonstrated that a reduction in contractile forces, via inhibition of actomyosin or culturing on soft substrates, paradoxically increased SREBP1 activation by altering Golgi membrane composition and DAG levels, ultimately enhancing SREBP1 proteolytic cleavage and nuclear translocation [61]. In our study, TiO_2_ nanotube substrates reshaped the local mechanical microenvironment of BMSCs. This may have induced compensatory mechano-chemical responses, including downregulation of LIPIN1 and subsequent accumulation and activation of SREBP1, despite a reduction in actomyosin tension [62]. These results suggest that cytoskeletal inhibition does not universally suppress SREBP1 activity, but may in some contexts—via organelle lipid remodeling—promote its activation through alternative mechanotransductive pathways.

Recent investigations indicate that the regulation of SREBPs by a soft matrix suggests that cellular relaxation can foster the lipidogenic differentiation of MSCs [63, 64]. In the study, inhibition of Srebp1 expression in the Flat group revealed that diminished lipid synthesis and uptake prompted BMSCs to favor osteogenic differentiation over lipogenic. Nevertheless, the precise molecular mechanisms underlying the impact of fatty acid metabolic remodeling on BMSCs differentiation necessitate further scrutiny. Achieving equilibrium between lipogenic and osteogenic differentiation is pivotal and warrants additional exploration.

In conclusion, TiO_2_ nanotubes have the capacity to modulate BMSCs differentiation via mechanical cues induced by actin polymerization contraction. Mechanistic investigations have revealed that mechanical stimulation enhances Lipin1 expression, leading to the inhibition of SREBP1 activation. Decreased expression of SREBP1 results in the suppression of fatty acid synthesis, thereby fostering osteogenic differentiation of BMSCs. The progress achieved in this study will serve as a significant reference for understanding the homeostasis of BMSCs differentiation within micro- and nano-morphological environments, while also offering novel insights into the interplay between mechanical signals from the ECM and fatty acid metabolism. Nonetheless, this study is subject to certain limitations, as no *in vivo* experiments were conducted to ascertain whether this mechanism elicits similar effects *in vivo*. Future investigations should explore the potential of TiO_2_ nanotube-shaped implants in guiding stem cell differentiation and mitigating abnormal bone remodeling.
